# Book Reviews

**Published:** 1833-03

**Authors:** 


### CRITICAL ANALYSES.

Quæ laudanda forent, et quæ culpanda, vicissim,
Illa prius, cretâ; mox hæc, carbone, *notamus.*—Persius.

*A Treatise on the Diseases of the Liver, and on Bilious Complaints; with Observations on the Management of the Health of those who have returned from Tropical Climates, and on the Diseases of Infancy.* By George Hamilton Bell, Fellow of the Royal College of Surgeons, Edinburgh; late residency Surgeon, Tanjore.—8vo. pp. 152. Bell and Bradfute, Edinburgh; and Longman and Co., London.

Diseases of the liver, not only in our eastern possessions, but even here, have gradually assumed a character of vast importance, and hence demand from the medical practitioner a corresponding degree of attention. Whether it be, from fashion, that hepatic disorders are more noticed now than formerly, or whether, as we suspect with our author, that they are really more prevalant than they used to be, we shall not attempt to determine: still we cannot but allow that "the supposition is not without plausibility, which avers that something like an intertropical tendency to liver complaints may have been imported into this country:" for, as our author observes,

"The sources of what are usually termed hereditary diseases may often be traced to some misfortune, neglect, or imprudence, in a predecessor. Thus the habits of living of a father, a grandfather, or even of some more remote ancestor, or a cold which he has neglected, may have engendered the gout, or the consumption, under which his descendants suffer. So it is well known that every one who has been much exposed to a hot climate acquires a predisposition to hepatic affections; and when we remember the number of our countrymen, or of their descendants, who annually return from the intertropical possessions of Britain, labouring under the diseases of the climate, and become fathers of families; or who themselves suffer during the remainder of their lives under the morbid affections which they have brought with them, we shall have no reason to be surprised at the diffusion and very general prevalence of diseases, which have not hitherto been regarded as indigenous in the temperate zones.

"It is also possible that the present habits of life in Great Britain, and in particular the increase and more general diffusion of luxurious living, may have produced a greater tendency to biliary derangements than naturally belongs to our climate. But be the cause what it may, it cannot be denied that the liver is daily becoming more prominent as a source of disease in this country: and no general medical practitioner, therefore, can safely be ignorant of the intertropical practice in hepatic disorders. In my own practice, since my return from India, I have derived great benefit from keeping my attention steadily fixed on the condition of the liver and duodenum; so much so, indeed, that I feel the less apology to be necessary for the present attempt to communicate to the profession the result of the observations on this class of diseases, which I have now had opportunities of making, both in India and in Great Britain." (*Pref.* vii.)

Such is the praiseworthy object of the present treatise, an object which its author has very ably achieved: for, though short, (all extraneous matter being excluded,) it is comparatively copious, and, though concise, it is (as far as an isolated treatise can be) complete.

We like the plain, straight-forward style in which this work is written; no mystification, no labouring after effect; but diseases are so graphically pourtrayed, that the descriptions bear the impress of truth, and carry conviction to the reader's mind that the writer is familiar with the topics on which he treats.

Dr. Bell's experience has led him to distinguish at least two kinds of acute hepatitis, which he denominates *sero* and *puro* *hepatitis;* and this distinction appears to be not only pathologically, but practically, important. The following are part of the "preliminary observations" on this subject.

"Such cases as the following have each received the name of acute hepatitis. An intertropical sportsman has been much exposed to fatigue; he has been snipe-shooting during the heat of the day, under an unclouded sun, and up to his knees in water. He is suddenly seized with excruciating pain in the right hypochondrium. As if struck with the sun, he is unable to move, and is carried to his quarters in a litter. He is found to be in a state of high fever; and his right side is so exquisitely painful, that even the weight of a single sheet is hardly endurable. Forty ounces of blood are drawn from his arm, and thirty leeches, followed by a large blister, applied to his side; and by pursuing a well-managed course of antiphlogistic treatment, he is in a few days restored to health—resolution has been accomplished. Or, if the result prove less favorable, there will remain weight and uneasiness in the side, and he will become a tropical valetudinarian; adhesion, in this case, having most likely taken place between the covering membrane of the liver and the peritoneal lining of the diaphragm, or abdominal walls.

"Again, a man is attacked with shivering, followed by feverish symptoms, which are relieved. His illness is pronounced to be a 'bilious attack;' but his tongue is white, his pulse tense, and his bowels irregular; and after eight or ten days he is again attacked with shivering. The case is supposed to be one of irregular ague, and he is treated accordingly. His countenance now assumes an earthy hue, and he is evidently in a state of bad health. But he is told that 'a course of tonics, and aperients, and change of air, will set all right.' He travels by easy stages to the sea-coast, becomes worse on the road, and, by the time he reaches the end of his journey, is obliged to confine himself to bed. His pulse is now stationary at 120, he has a clammy skin, a dirty soddened tongue, his bowels are in great disorder, and although he probably has severe spasmodic twitches in the abdomen, there is nowhere any fixed pain. This goes on for some days, the patient settles down on his back in bed, his skin becomes clammy and wet, he falls into a state of low delirium, and dies from effusion in the brain. An abscess containing some pints of matter is found in the centre of his liver.

"It is cases such as these, and every practitioner must have met with them, which have led me to the conclusion that inflammation, when seated in diaphanous membranes, or in parts in which the healthy circulation consists of colourless blood, differs in symptoms, course, and termination, from the same affection when it occurs in parts in which the vessels, when in health, convey red blood. The distinction I regard as of sufficient practical importance to justify the subdivision of phlegmonous inflammation into what may be termed sero-phlegmon and puro-phlegmon; to which may be added muco-puro-phlegmon; the first making its attack on naturally colourless parts, and possessing all the characteristics of inflammation, viz. unnatural redness, heat, pain, and swelling, and having a tendency to end in effusion of serous fluid; the second being that affection in those vascular parts in which, the healthy circulation being already red blood, there is little or no apparent change, no great increase of heat, in which the pain when present is obtuse, and where there is a tendency to the deposition of pus; and the third attacking mucous surfaces, in which there are pain, heat, swelling, and unnatural redness, terminating in change of secretion and ulceration." (P. 7.)

"The symptoms, then, of sero-hepatitis are acute pain, referable to some part of the surface of the liver; pain over the clavicle; difficulty of breathing; dry cough; high fever; great thirst, and irritability of the stomach and bowels; urine high coloured, depositing a lateritious sediment; with pain much increased on pressure, and by attempts to lie on the left side." (P. 16.)

"*Treatment.* It is with a view to this, the most important object in an inquiry into the nature of disease, that I have ventured to propose that a line of distinction should be drawn between inflammation of the coat of the liver, and inflammation in its substance. For it seems to me that a due regard to that distinction may enable us to reconcile the difference of opinion which exists among good practitioners as to the treatment of acute hepatitis.

"The sanative effects of mercury in functional affections of the liver, and in diseases within its substance, may be regarded as established; but many physicians, particularly in this country, are of opinion that mercury ought not to be exhibited in acute hepatitis: and, so far as my own experience goes, I have not found it necessary, in sero-hepatitis, so long as the disease is acute, to put the system under the influence of mercury. I have reason, indeed, to believe, that much harm may arise from 'pushing mercury' in cases in which the acute inflammation is confined to the covering membrane of the liver; and of this the following case supplies an illustration.

"Several years ago, while in India, I was called to a station at some distance from my own, to see a civilian of high rank, who was considered by his medical attendant to have fallen into a state of great danger from an attack of hepatitis. This patient had been about twenty years in India. I found him in a state of low delirium, with an alarming tendency to dosing; his pulse was upwards of 120, thrilling and weak; his face swollen to double its natural size; and his mouth, throat, and tongue, in a terrible condition from ptyalism. I was told that he had been suddenly seized with excruciating pain in the right side, with fever, and all the other symptoms of acute hepatitis; that he had been very largely bled generally; that local depletion with leeches had been carried as far as possible; and that he had also been blistered. By those means it appeared that the pain had been completely removed from the side. Calomel had likewise been administered to a great extent; and the mouth had suddenly become affected, attended with great pain. The swelling of the mouth, tongue, and throat had increased to the state in which it was when I first saw the patient; but the pain had suddenly ceased, and delirium and comatose symptoms had supervened.

"The hepatic affection having been thus to all appearance mastered, the dangers now were sphacelation of the mouth and throat, and cerebral effusion. We therefore turned our whole attention to the head and circulation. But every attempt to lower the pulse failed, and the delirium, though it intermitted, was not manageable. The patient sunk and died; and, on examination after death, the liver was found perfectly sound. The inflammation had been overcome by the decided antiphlogistic practice pursued; and although the exhibition of mercury was according to rule, the case was not, in my opinion, one which called for the exhibition of mercury as a sialogogue; and the patient had perhaps been too long exposed to an Indian climate to admit of even the necessary depletion with safety, far less the deleterious effects of 'pushing large doses of calomel.'

"In the treatment of sero-hepatitis, it is not necessary to put the system under the influence of mercury. The extreme irritability of the stomach may render the exhibition of a scruple of calomel necessary; and this medicine is valuable in combination with purgatives. But salivation is in my opinion uncalled for, and may, as in the above case, become positively injurious in acute sero-hepatitis.

"The objects to be attained are, 1st, to weaken the general force of the circulation; 2d, to allay the irritability of the stomach; 3d, to overcome the morbid condition of the capillary vessels; 4th, to act on the bowels; and, 5th, to equalize the circulation, and allay morbid irritability." (P. 17.)

Local bloodletting is much recommended by Dr. Bell, and seems, in Indian constitutions, to be often a more safe and efficient practice than general depletion. The following, however, may not be a useless caution to English practitioners, on their first arrival in India.

"In India, leeches are a most efficacious means of accomplishing depletion; for the tropical leech is not only so large as to be capable of containing from one to two ounces of blood, but it leaves a wound more like that of a bayonet than a leechbite; so that a dozen or twenty leeches will not only draw a great quantity of blood, but will do so with wonderful rapidity,* Having, therefore, bled freely with the lancet, exhibited a large dose of calomel, &c,, a dozen or twenty leeches ought to be applied over the region of the liver, and their removal followed by a lengthened fomentation." (P. 22.)

"*Puro-Hepatitis.*

"*Symptoms.* The condition of the hepatic vessels which leads to suppuration in the substance of the liver, seems to be so little different from their usual state (at least so far as is indicated by symptoms), that very frequently the first intimation which a patient has of serious disorder of the system, is what is too often to be reckoned proof of the formation of an abscess. He is attacked with a shivering fit, which is followed by an irregular hot stage, ending in profuse clammy perspiration. Even after this there may be no symptoms pointing out the destruction which is going on in

* "A very dangerous case of exsanguination occurred in the Indian practice of a young friend of mine. Accustomed to the leeches of a London hospital, he had ordered the applicatlon, to the knee of a robust man, who had suffered a fall from his horse, of as many leeches as would adhere. Upwards of forty of the large Indian leeches were applied, and before one had dropt off the man fainted, and could scarcely be reanimated."

his liver. The patient suffers from irregular feverish symptoms, and has the impression that something very wrong is taking place; but neither he, nor probably his medical attendant, is aware that he is stricken with a mortal malady. As the case advances, there are occasional severe shivering fits, and distressing night-sweats, the pulse rises, the tongue is furred, and, from the appearance of the patient's countenance, it is evident that he is labouring under some great internal disease. Still there may be no symptom referable to the liver; great derangement of the bowels ensues, and there is much suffering from dyspeptic symptoms. In some instances there are severe spasms in the diaphragm, and violent tenesmus. After some days (or it may be even weeks) the patient is attacked with low delirium, and dies as if from effusion in the brain. This is an extreme case.

"In the less obscure cases there are fulness, weight, and uneasiness in the right side, increased on pressure. There is pain in the right shoulder, or in the back; there is a dry cough, the stomach is disordered, and the bowels much deranged; and though the pulse may not be materially affected, there are alternate aguish and and feverish symptoms. There is urgent thirst, the tongue is furred, and the urine high-coloured, depositing a lateritious or pinky sediment. There is great risk that these symptoms will soon be followed by decided indications of the formation of an hepatic abscess.

"Cases occur in which very extensive abscesses have appeared to form very rapidly, death ensuing within eight or ten days after the accession of symptoms of acute sero-hepatitis, and in which it has been found, on the post-mortem examination, that one or more extensive abscesses had formed in the substance of the liver. I am persuaded that in all such instances the abscesses had formed insidiously and unnoted; and that the symptoms which had forced themselves into notice were those produced by the inflammatory action having extended itself to the peritoneal covering of the liver.

"In other instances the hepatic affection has been first detected by the patient finding that he could not endure the weight of his watch in his waistcoat pocket, or that the pressure of a friend's hand while leaning on his arm has produced a sickening uneasiness. Such slight symptoms of morbid sensibility in the region of the liver have been soon followed by melancholy evidence that suppuration had taken place in that organ." (P. 30.)

Of the insidious advances of this form of disease, several very instructive illustrations are detailed; but at these we can only slightly glance.

"Mr. Assistant-surgeon H—, a stout man, about thirty-five years of age, arrived at Tanjore on the 25th of March, 1826, in attendance on the late Bishop Heber. He went to church on the following day, Good Friday, made a round of calls, and dined at the Residency. On calling for him on the forenoon of the 27th, I found him confined to his sofa. He told me he had a slight feverish attack, but that he expected his usual dose of oil, which he had taken, would put him to rights again. As his hand was hot and dry, and his pulse quick, I advised him to take some tartar-emetic solution. He objected to this, but agreed to take a dose of compound powder of jalap. When I saw him in the evening, I found him still feverish; his bowels had been opened, bnt the evacuations, though apparently bilious, were fetid and unwholesome. I prescribed effervescing draughts, and gave him at bedtime six grains of calomel with some James' powder, prescribing the black draught in the morning." (P. 32.)

The treatment during some days being chiefly a combat with symptoms, no accurate diagnosis being made, we pass on to the closing scene.

"4th April. I sat up with Mr. H. the greater part of the night. I continued to administer calomel combined with opium, of the latter of which, in the course of the night, he had four grains; and I also gave him, in hopes of keeping down the pulse, thirty drops of the tincture of digitalis in different doses.

"Mr. H., though prepared for death, thought that he had been reduced in former illness to a state of more imminent danger, and was persuaded that he owed his life to the perseverance of his then medical attendant, in giving him wine and nourishment; and he requested me to act in a similar manner. I therefore continued, until he could no longer swallow, to administer wine, &c. Mr. H. retained his consciousness until two o'clock in the afternoon, and died a little after four.

"*Sectio cadaveris.* An abscess, containing upwards of a pint of matter was found in the great lobe of the liver, on the point of bursting into the abdomen. It was seated nearly in the centre of the lower surface of the lobe. On the upper surface of the great lobe there was an old adhesion of considerable extent between the diaphragm and the liver. The gall-bladder was full of green-coloured bile. The bowels throughout showed that there had been much irregular spasmodic action in them. The cardiac end of the stomach was full; the pyloric end was contracted. The duodenum was also contracted. These parts may have been affected by the pressure of the abscess on them.

"In this case it is remarkable, that the patient, a man of great judgment and perfect coolness, who was thoroughly persuaded that he understood the nature of his illness, never suspected disease in his liver. He said that in a former illness an adhesion had taken place between the diaphragm and the lungs, and to this he ascribed the spasms in the diaphragm. I examined the chest, and found that the pleura were attached almost throughout their surfaces. The adhesion of the liver to the diaphragm will, however, better account for the symptom in question.

"Although throughout my attendance on Mr. H. I feared that some great internal organic lesion existed, I could not decidedly say what it was. The absence of pain in the right side or shoulder, the patient's being able to lie on either side, and his not confessing to his ever having had any rigors, appeared to me almost like proof that there was no abscess in the liver; and I confess that I went on very much in the dark; administering to symptoms as they occurred, and most anxious to credit Mr. H.'s assurances that he had suffered in the same way in former illnesses.

"I have since ascertained, that, three weeks before my attendance on this gentleman commenced, and previously to his having marched upwards of 200 miles down the Coromandel coast of India for ten days, during the hottest season of the year, he had suffered at Madras from repeated attacks of shivering. There can be little doubt that these symptoms marked the formation of matter. And it adds considerably to the interest of this fact, that so little were those shivering fits connected, in Mr. H.'s mind, with his fatal illness, that, in our many conversations on his case, he never once alluded to them. I may mention, however, that an intelligent officer, who commanded the bishop's escort, observed to me, before the disease assumed a serious character, that, from the appearance of Mr. H.'s countenance on the march, he felt convinced that he (Mr. H.) had some mortal malady hanging about him." (P. 37.)

The following likewise we should be scarcely justified in omitting.

"It happens very fortunately for my present object, that I have lately had an opportunity of watching (in the case also of a medical man) the progress of puro-hepatitis to its fatal termination in this country.

"Mr. Surgeon B., after having served eleven years in India, was compelled by the state of his health to return to England. His mode of life, during the latter years of his service in that country, had been exceedingly intemperate, and latterly he had lived almost entirely on ardent spirits. The consequence had been alarming disorder of the stomach, which, before he left India, had assumed the character of organic disease. His health improved on-board ship, where he had been enabled to wean himself from the brandy-bottle. In August 1830, while in the west of England, he was attacked with what was considered by his medical attendant as irregular ague, for which he was treated in the usual way. But, although the aguish symptoms were relieved, he was not restored to the health he had enjoyed on his landing in England. His stomach again became irritable, and he rapidly lost flesh. He came to Edinburgh in the beginning of December 1830, and, when he placed himself under my care, the following was his condition: He appeared to be in the last stage of general atrophy, and his countenance bore the indescribable marks of a man sinking under organic disease. His principal suffering was in his right leg, which was bent up upon his body, this being the only position which gave him any thing like relief. His skin was cool, his pulse 120, his tongue, though not loaded, had the fleecy-grey moist look which betokens organic disease; and he had great irritability of the stomach, with difficulty of swallowing, and occasionally severe pain, referrible to the cardiac orifice of the stomach. He had frequent attacks of hiccup, which sometimes lasted for hours; and there were occasionally acrid sour eructations. His bowels were open, but the alvine discharges were unnatural in appearance. He told me that, although there was no symptom referrible to the liver in the intermittent feverish attack with which his present illness commenced, yet so impressed was he with the belief that there was something wrong in that organ, that latterly he had persuaded his medical attendant (although he thought it unnecessary) to apply leeches, and to blister him on the right side. He had since then resorted almost constantly to blisters over the stomach, and the only quiet sleep he had enjoyed for months had been during their vesication.

"I told Mr. B. that there was every reason to fear that an abscess had formed in the liver; that there was disease at the cardiac orifice of the stomach; and that I could account for the peculiar suffering in his leg only by supposing that there must be a tumor within the pelvis pressing on the nerves of the leg.

"He lingered on for seven weeks with little change of symptoms; his pulse varying from 120 to 140: his stools, although unwholesome in smell, were latterly excrementitious, and not ill-coloured: urine in very small quantity, and thick: occasional troublesome cough, with much expectoration. There was almost constantly severe suffering in the right leg and hip. Sleepless nights, and colliquative sweats.

"My treatment consisted of aperients and anodynes, a succession of blisters over the stomach, and anodyne liniments to the spine. Mr. B., would have nothing to do with mercury in any shape.

"*Sectio cadaveris.* Extraordinary emaciation. There was much flatus in the alimentary canal; the omentum adhered to the peritoneum lining the abdomen and the floating viscera at several points. The liver adhered to the peritoneal lining of the diaphragm and abdominal walls throughout its whole convex surface, apparently the consequence of some former attack of hepatitis. The whole of the viscera on the right side formed a general mass of disease. The posterior portion of the great lobe of the liver was gangrenous. On attempting to raise the liver, the fingers broke into a large abscess, which seemed to occupy the whole of the lower part of the great lobe, and was filled with matured pus. The ascending colon, the duodenum, and liver, were formed into one mass of disease; and, on opening the stomach, the whole of its inner coat was found to be thickened and ulcerated in various parts. The pylorus was hard and almost impervious. The mucous coat of the cardiac orifice of the stomach, and of a portion of the œsophagus, was thickened, ulcerated, and black. The coats of the duodenum were thickened and black, as were those of the ascending arch of the colon. The right kidney was involved in the disease; and the most extraordinary point of the dissection was, that the right ureter had been cut off from its connexion with the bladder; and there had been formed an immense sac of urine behind the peritoneum, and running down among the muscles like a psoas abscess. The psoas and iliacus internus muscles of the right side were black, and apparently mortified.

"This gentleman must have carried a great portion of this extensive disease along with him from India, and the abscess of the liver must have existed for six months.

"These cases are, I think, sufficient to illustrate the obscure nature of the symptoms of puro-hepatitis; and I regard them as the more valuable, because the sufferers were both of them intelligent medical men, who had the natural bias of Indian practitioners to direct their attention to every feeling referrible to the liver." (P.39.)

Counter-irritation, either by the actual cautery or by caustic issues, Dr. Bell speaks of with much confidence, as among the most likely means of preventing the formation of abscess in the liver, and of favoring the absorption of the matter when formed; of which fortunate result several important cases are on record.

The chapters on "Chronic Sero and Puro-Hepatitis," "Functional Derangements of the Liver," "Jaundice," the "Disorders of Tropical Valetudinarians," Diseases of Children," "Irregular Complaints of Infancy," and "on the age at which Children born in India should be sent to Britain, and on the Management of their Health in this country," contain many valuable remarks; but for these we must refer our readers to the volume, which we recommend to the perusal not only of the members of the medical profession, but of the public, and more especially to the attention of practitioners and others who are either themselves going to, or have any connexions in, the East.

---

*A Further Examination of the Principles of the Treatment of Gout; with Observations on the Use and Abuse of Colchicum.* By Sir CHARLES SCUDAMORE, M.D., F.R.S., &c. &c. *The second Edition, considerably altered and enlarged.*—8vo. pp. 127. Longman and Co., London.

IN this book Sir CHARLES SCUDAMORE offers many observations respecting the effects of certain remedies frequently used in gout, which patients who are afflicted with the disease, and who are inclined to trust to their own rather than their physician's skill, will do well to attend to, and from which the medical practitioner may himself derive some very useful hints.

The chief object of these pages, we are told in the preface, is to inquire into the real merits of the Colchicum autumnale as a remedy in gout. This subject may appear to be almost exhausted; but those who have opportunities of observing the injudicious and even dangerous manner in which this powerful and much-abused remedy is used by patients themselves, as well as the erroneous principles upon which it is too frequently employed by the profession, will agree with us that Sir Charles makes no unprofitable demand upon our time, when he calls our attention to a "further examination" of its powers, and to the particular kind of cases in which it may be safely and advantageously employed. If we are much indebted to those who *add* to our list of remedies, our thanks are also no less due to those who endeavour to establish the *real* merits of medicines which have been long known, but which have been so indiscriminately employed as to render it extremely doubtful to what degree of confidence they are fairly entitled, and in what manner and in what particular class of cases they can most usefully be employed.

In the treatment of gout, the practitioner must steadily keep in view that the peculiar external appearance of inflammation, which we denominate the gout, is the least part of the disease, and is to be regarded as the sure sign of some error in the constitution, which is the real disease to be principally treated; the local suffering being the effect of a cause existing in the system. Sir Charles believes that the gout, which appears to the eye as an external disease, is most essentially depending on that species of repletion which belongs to the vessels of the abdominal viscera, and chiefly of the liver.

"In its progress, it manifests this connexion more strongly; and I do affirm that, in every long-established case of gout, the functions of the liver are more or less unhealthy, in combination with a disordered condition of the intestinal canal, with evidences of error in the secretions derived from the kidneys and from the skin; and, also, in proportion as the tyranny of the disease becomes established, the nervous system partakes largely in the derangement. The stomach itself, which at the earliest periods of gout is often little, or not at all, affected, now becomes disordered, and serious indigestion commonly prevails." (P. 7.)

We pass over the "short review" which is given "of the theory and practice which have prevailed in ancient and modern medicine up to the present time:" it is a learned but somewhat useless episode.

From various experiments which he has made, Sir Charles infers that the eau medicinale, Wilson's tincture, and Reynolds' specific, are all preparations of colchicum.

"In direct opposition to the results of Sir Everard Home's experiments, I found that the sediment deposited by the wine of colchicum, or by his own infusion, consisted chiefly of mucilage and extractive matter, and that it was a perfectly inert substance administered as a medicine. I derived the same result, however, as he experienced in regard to the sediment deposited by the eau medicinale, which acted powerfully; and hence the great difference of operation as a medicine, whether the eau medicinale be administered when poured off clear from its sediment, or with the sediment shaken up. The same dog recovered completely from a dose of the clear liquid, while an equal quantity of the turbid proved fatal in nine hours. I suspect that the composition of this extraordinary medicine may be the inspissated juice of the fresh roots, purified and much concentrated, mixed with a light French wine." (P. 33.)

Three drachms of the eau medicinale, and six of Wilson's tincture, given each in two doses, proved fatal to a dog; and all the strong preparations of colchicum produced the same effects, both as to the symptoms and the morbid appearances found on dissection. But three ounces of the *acetum colchici*, mixed with magnesia, administered in two doses, did not occasion illness, but merely acted moderately on the bowels and kidneys. This fact is offered in proof of the mildness of this preparation, " and whether or not it be useful or efficacious as a medicine, must be determined by its effects on the human subject."

Sir Charles objects to any countenance being given by the regular physician to the nostrums just mentioned, from having long observed their very injurious effects, administered as they are in a manner calculated rather to suppress than cure the disease, and, if taken with frequency, in a serious degree to injure the nerves of the stomach, its mucous membrane, and that of the intestinal canal; impair the functions of the liver, and debilitate the powers of the whole system. When the medical practitioner objects to the use of any empirical remedy, the public are too ready to believe that he is influenced by interested motives alone, and that he is much less apprehensive that his patient will suffer in his constitution, than that he himself will " suffer in pocket," by a diminution of his fees. But we are much mistaken if the confidence of the public is not now greatly shaken with respect to the safety of either of the nostrums referred to. We have often been told by gouty patients that they could relieve the local pain of the disease by Wilson's or Reynolds' remedy, but that they were afraid to have recourse to them, from a conviction of the mischief they had inflicted upon their general health.

Ample experience has convinced Sir Charles of the impropriety of opposing the threatened invasion of a fit of gout by any preparation of colchicum; for, however unwise and unnecessary it may be to allow the symptoms, when formed, to pursue their own tedious and painful course, it is too much of a contrary principle to thwart nature's design altogether, by seeking in this manner to oppose the production of the fit. To illustrate this opinion, some cases are briefly related, in which colchicum arrested the local pains, but greatly increased the constitutional ailments, and appeared to lead to organic disease.

" Having delivered so strong a protest against the inconsiderate employment of the strong preparations of colchicum, the question will be fairly proposed, is this medicine, in any of its forms, a proper remedy to be employed in the treatment of a fit of the gout? In my answer, I trust that I shall not be found inconsistent, if I recommend a cautious employment of the mildest preparation of colchicum which we possess, the acetum, either combined with calcined magnesia and sulphate of magnesia in some pleasant vehicle, or with carbonate of magnesia and the sulphate in a saline draught, (as I have fully described in my Treatise,) repeated three or four times in the twenty-four hours, during the violence of the symptoms, and afterwards in a more occasional manner, always taking care not to irritate the stomach by nausea or vomiting; an effect, however, which scarcely ever happens from this combination of ingredients. I make it my object to seek the slightest, instead of the greatest, aid to the removal of the symptoms from colchicum. The difference is extreme between the administration of a strong preparation of colchicum, per se, so that it may exert all its power on the stomach and nervous system; and the taking of a preparation so mild as the acetum, used in conjunction with corrective and gentle aperients; by which method it is not long retained in the stomach; while the leading object of the treatment is to evacuate freely, yet not severely, the morbid secretions; adding, too, the important influence of mercurial alteratives, the pilula hydrargyri or calomel, according to the indications to be fulfilled; and probably combined with James's powder, and occasional doses of compound extract of colocynth. In the majority of cases, all the severe symptoms of gouty inflammation and pain yield most favorably to this method of treatment, joined with anodyne preparations and sudorifics at night; and, also, with the auxillary influence of careful local treatment; all of which methods I have set forth in my treatise. The most difficult and untractable cases are those in which some one or other of the strong preparations has been the oft-tried remedy, breaking the force of the acute symptoms, but leaving a disposition to chronic gout in its most harassing forms. The habit of relapse becomes established in the constitution. The strong medicine is not a means of cure; the weaker one is incapable of affording the same prompt and certain relief which it scarcely ever fails to do, when such false practice has not been pursued. But no injury, present or future, awaits the proper employment of the acetum colchici draught, or of the acetous extract, of which I shall have future occasion to speak. I have been frequently called upon to undo the serious evil which has been committed by the empirical treatment; and by perseverance I have succeeded." (P. 55.)

Gout is frequently complicated with other maladies; but, whatever the existing disorder may be, it should be treated, with very little modification, according to the actual symptoms, and not upon any fanciful theory regarding the gout. Sir Charles observes, that ammonia, if given in large doses, possesses a higher power than any other medicine of exciting the gouty disposition into action. He considers it also an axiom of importance, that mercurial medicine should never be administered to gouty persons to any extent which carries with it the risk of producing mercurial fever.

"Before I take leave of the subject of the treatment of a paroxysm of gout, I wish to deliver my sentiments more at length respecting colchicum, and to consider the merits of opium as an auxiliary remedy. I venture to assert, as a principle, that, in the administration of colchicum, it should be our care to use it with a most sparing and cautious hand, viewing its effects rather as palliative than curative, as subordinate to the more radical means of treatment rather than as the chief agent for the cure of the disease. As I have already had occasion to observe, the acetum colchici is the mildest of all the preparations. The Pharmacopœia directs one ounce of the fresh roots to seventeen ounces of fluid, sixteen of diluted acetic acid, and one of proof spirit. For the vinum colchici (so called) the proportion of the fresh root is twelve ounces to twelve ounces of fluid, four of proof spirit, and eight of water. But, besides this extraordinary difference of strength, arising from the different quantities of material in the two preparations, we may consider that the acetic acid exerts a modifying power over the active principle of the colchicum (veratrine), as it does over opium and squills, rendering its action on the animal economy milder. When the acid is neutralized by an alkali, the colchicum still remains in solution. When desiring the most cooling form of draught, I give the acetum with a dose of neutralized lemon-juice and carbonate and sulphate of magnesia; and, when inflammatory action is more than usually active, I add tartarized antimony. If wishing more distinctly a purgative effect, I substitute the calcined magnesia for the carbonate, omit the neutralized lemon-juice, and probably add some tincture of senna or a small portion of tincture of jalap. In most instances, and especially where the empirical treatment has not been before employed, the continued use of this draught fulfils, towards the removal of the gouty symptoms, all that a prudent physician should desire to obtain from colchicum. I sometimes prefer the administration of the acetum colchici reduced to the solid form, and in a pill. The idea of making a preparation of this kind occurred to me some years ago, when I requested Mr. Garden, of Oxford street, to evaporate the acetum colchici down to the consistence of a soft extract, over a water-bath, at so low a temperature as to be free from all risk of decomposing the material; in which state it may be considered that one grain is equivalent to eighty minims of the fluid. I commonly direct two grains of the subcarbonate of ammonia to be united with one grain of this extract; conceiving that the medicine agrees better with the stomach when the acid is neutralized. It may be joined with sedative ingredients, as Dover's powder, or conium, or hyosciamus; or with a purgative, as the compound extract of colocynth, or extract of rhubarb, as circumstances shall direct; or it may be given alone." (P. 88.)

The acetum colchici is in use at several of our hospitals in cases which require a moderate influence of colchicum; and it may be added that Sir Henry Halford mentioned it with approbation in a paper on Gout which he read to the College of Physicians in June 1831.

Sir Charles notices a new preparation of colchicum which comes from Mr. Battley. This is the inspissated juice of colchicum, obtained by expressing the fresh roots, and which, by a certain process, is purified and carefully concentrated to the consistence of an extract. Dr. Hue, of St. Bartholomew's Hospital, gives the following opinion of its virtues:

"'I have so often witnessed the very uncertain* and variable effects of the wine of the roots, and of the seeds of colchicum, and more particularly of the acetum, that I was on the point of trying this very valuable medicine in substance, when I met with what I was looking for in the inspissated juice, an unsophisticated preparation. I have given it a most extensive trial, with a success which has exceeded my most sanguine expectations. In acute rheumatism, the most severe symptoms have given way to the medicine in not longer than twenty-four or thirty-six hours after its exhibition; and the proportion of favorable cases is so great as to justify the strongest terms which I might be disposed to employ in recommending it. I have less experience of it in gout. The dose of the preparation is one grain every four hours; and, as soon as it may have produced either sickness or purging, I give it only every six hours, or even at longer intervals, or altogether withhold it.'" (P. 92.)

Sir Charles states, that, in the treatment of acute rheumatism, there is far less objection to the employment of colchicum, so as to bring the system decidedly under its influence, than in the treatment of gout; for the rheumatic inflammation is very much less a secondary disease than that of gout; nor is acute rheumatism a disease of certain return, like gout.

The baths at Buxton are highly spoken of in cases of chronic gout or rheumatism. Upon the diet and regimen which the gouty patient requires, and upon preventive remedies, some good practical remarks are made.

We have now laid before our readers the leading points which are discussed in this brief work. It is written in rather a desultory manner, but its perusal will not occupy more than an hour, and cannot fail to furnish the practical reader with some "fit food for his memory." The directions for the use of any remedy that is mentioned are open and candid; and we are very happy to find that Sir Charles "reprobates mysterious conduct, and, on the contrary, considers that the physician should always be ready to explain clearly his views and principles of treatment."

* "In the course of my experiments, I found much difference in the goodness of the colchicum-roots, some being very porous and spongy, and absolutely inert in quality. Mr. Battley pronounces the following as the signs of a good root: 'roundness, plumpness, firmness on cutting, and being covered with a creamy matter immediately on being incised. The most fit time of the year for taking up the root is in June, before the offset from the parent root is formed.' The best roots are procured from Oxfordshire and Buckinghamshire."

*Illustrations of Elementary Forms of Disease.* By Robert Carswell, m.d., Professor of Pathological Anatomy in the University of London, &c. *Fasciculus I. Tubercle.*—Imperial 4to. London, 1833.

Of all the sciences connected with medicine, pathological anatomy, or the study of the changes produced by disease, remained for the longest period uncultivated, and even disregarded. The attention of the Hippocratic school was almost exclusively directed to the symptoms of disease; and it is vain to expect that the followers of the brilliant medical theories, which sprung up one after another during the seventeenth and eighteenth centuries, would think of pathological investigation: their supposed knowledge of the proximate cause of disease rendered all further inquiry needless and unnecessary; and to the anatomist alone we are indebted for any pathological discoveries made during this period.

In a science like pathological anatomy, which could be founded only by the accumulation of an immense number of well-conducted observations, much time necessarily passed before any attempt was made to arrange them in systematic order; and, indeed, we find that, down to a very recent period, all works on this subject have been confined to the mere detail of isolated facts.

To Bichat we are certainly indebted for the first attempt at classification; and, since his time, the labours of succeeding pathologists, both in this country and on the continent, have been continually furnishing materials for, and contributing to, the formation of a more perfect system of Pathological Anatomy. Baillie, Abercromby, Hooper, Bright, Boyle, Laennec, Andral, Louis, Cruveilhier, and many others, have each taken up the study of certain organs, or classes of organs, and, by their laborious researches, have thrown such light on their respective subjects, that it only required the skilful hand of one well acquainted with their labours to form them into a system, and elevate the study of morbid anatomy to the rank of a science.

Various attempts of this kind have been made; but they have certainly fallen far short of what might have been expected. Instead of studying pathological anatomy for itself, some authors have taken physiology as the basis of their classification, and, in their attempt to submit the latter to the laws which regulate the former, they have encumbered it with a mass of theory and hypothesis, the necessary result of the imperfect state of that science on which their arrangements are founded. Others, viewing pathology through the medium of general anatomy, have committed faults of no less magnitude; for, as we have the same morbid process affecting different tissues, it is utterly impossible, by this *arrangement*, to avoid continual repetitions, or preserve order or precision.

Works, therefore, founded on such artificial systems, though often rich in facts and observations, are extremely ill adapted for those who wish to learn the elements of this science; the materials being accumulated in such confusion as speedily to tire and disgust the student. A philosophical system of pathological anatomy should rest upon the general facts naturally arising out of its own laws, and not be made dependant for its foundation upon those of any other science. Every morbid change must be studied for itself, as it appears in each tissue and in each organ, in the solids and in the fluids, and its own specific characters pointed out and distinguished amidst the accidental circumstances with which they may be combined.

Entertaining such views, it gives us great pleasure to see this important subject taken up in a proper manner by Dr. Carswell, a pathologist of distinguished talents, whose whole life appears to have been almost exclusively devoted to the study of the subject, and whose extensive opportunities of acquiring a knowledge of it have only been equalled by his zeal and industry in taking advantage of them. He adopts a purely "*Pathological arrangement*, making diseases themselves the basis of his classification. In accordance with this plan, each diseased state or product will, in the first place, be represented under that form which may be considered as constituting its type, or most perfect state, that is, that peculiar assemblage of characters under which it most generally presents itself, and by means of which it is distinguished from other diseased states; and, in the second place, as it appears in the different tissues, systems, and organs of the body. The essential characters of the disease, and its various modifications and varieties, will be thus successively depicted, before passing to another and a different disease."

In thus taking disease itself as the basis of his arrangement, Dr. Carswell has adopted the only true ground upon which a clear and faithful view of his subject could be exhibited: he will do, in short, for morbid structures that which Bichat did for the healthy tissues, and with the guarantee for the further execution of his work which the first fasciculus affords, we do not hesitate to affirm that Dr. Carswell's work will form as remarkable an epoch in the history of pathological as that of Bichat did in general anatomy.

Our author's views are also calculated to lead the way to great improvements in the practice of medicine; for, considering those changes found at death rather as the effects of disease than as disease itself, he has not confined himself, like the generality of morbid anatomists, merely to the accurate description and delineation of these ultimate changes, but has endeavoured to follow each up to its source; and the attention of the physician, being thus chiefly directed to the primary changes in healthy structure, he will be enabled to apply his remedies with a precision and effect hitherto unknown.

Dr. Carswell has chosen *Tubercle* as the first subject for illustration, "because this morbid product affords peculiar facilities for developing the plan by means of which he intends to illustrate the elementary forms of disease." He defines tubercle to be "a pale yellow, or yellowish-grey, opaque, unorganized, substance, the form, consistence, and composition of which vary with the nature of the part in which it is formed, and the period at which it is examined."

Our author then goes on to describe, under different sections, the seat, form, consistence, colour, composition, progress, and termination of tubercle.

*Seat of Tubercle.* The most ancient opinion with regard to the seat of tubercle is that of Sylvius, who placed it in the supposed lymphatic glands of the lungs; and this notion has been revived in our own day by Broussais. A much-more-generally-adopted opinion places it in the cellular membrane of the parenchyma of organs. This opinion was first broached by Stark, and adopted by Baillie, Bayle, and several others. Laennec, Andral, and Louis, while they give this as its most general seat, admit, at the same time, its occasional formation on the surfaces of mucous membranes. Cruveilhier, on repeating some experiments first made by Clayton, above one hundred years ago, and since by Saunders, in which tubercles were formed by the injection of mercury into the veins and trachea of dogs, was led to maintain that tuberculous matter was a sort of pus modified by the seat and nature of the inflammation, and that it was formed on the surface of mucous membranes, and more particularly in the air-cells of the lungs. Dr. Carswell, taking a more general and less exclusive view, thus explains his ideas on the subject, "considered in a general point of view, and in relation to the different tissues, systems, and organs of the body, the mucous system is by far the most frequent seat of tuberculous matter. In whatever organ the formation of tuberculous matter takes place, the mucous system, if constituting a part of that organ, is in general either the exclusive seat of this morbid product, or is far more extensively affected with it than any of the other systems or tissues of the same organ. Thus, the mucous system of the respiratory, digestive, biliary, urinary, and generative organs, is much more frequently the seat of tuberculous matter than any other system of tissue which enters into the composition of these organs."

We think this discovery of more importance than might at first be imagined; for tuberculous matter being once demonstrated to be a depraved secretion, taking place, for the most part, on the surface of mucous membranes communicating with the exterior, we are not only directed in the application of remedial and preventive means, but have even grounds to hope that some remedy may yet be found for this fatal disorder.

The various circumstances which modify the form, consistence, and colour, of tuberculous matter, are clearly pointed out by our author. When speaking of the composition of tubercles, the theory of Dupuy and Baron concerning their hydatid origin is shown to be inconsistent with the fact "that tuberculous matter is generally formed *ab origine* on the secreting surface of hollow organs."

Softening of tubercle is shown not to proceed from the centre to the circumference; and the appearances which led Laennec and others into this error, are very ingeniously explained. In like manner the erroneous ideas respecting encysted tubercle are pointed out.

Under the head of *terminations of tubercle*, the author's opinions on the important questions of the absorption of tubercle, its transformation into calcareous concretions, and osseous and cartilaginous tissue, of the filling up of caverns, and the obliteration of bronchi, are fully and clearly stated, and are of the greatest importance in a practical point of view.

The delineations which illustrate each point relative to the disease are executed in a style which far surpasses any thing that has yet appeared in this country or on the continent. They are drawn on stone, by the author, and are quite free from the high colouring which may be noticed in most plates of the kind hitherto published. They represent, moreover, the morbid parts and the portions of the organs in which they are seated, of the natural size; a circumstance to which we attach great importance, as it enables the student to form correct impressions on the subject.

In plate I. a minute and almost microscopical dissection shows the tuberculous matter filling the bronchi, and traced from them along their last ramifications, even into the air-cells themselves. It has, no doubt, been by careful dissections on this plan that our author has been able to detect the real seat of tubercle, which could have no more been done by the usual transverse sections of the lungs, than could the minute anatomy of the brain have been discovered by the horizontal sections which for so long a period were the only means employed for demonstrating its structure.

The same matter is then represented successively in the uterus and fallopian tubes, in the kidney, the testicle, the gall-ducts, the lymphatic vessels, the follicules of the intestines, in the brain, in false membranes, and in blood itself contained in the spleen. The ulcerations of the vagina and intestines are also beautifully represented.

In plate IV. we find the various termination of tubercle, the contraction of caverns, the obliteration of bronchi, &c. But fig. 3 of this plate is, perhaps, one of the most interesting; we here see the manner in which hæmoptysis is produced, by masses of tubercles preventing the return of the venous blood from the extreme parts of the lungs, and thus leading to effusion upon the surface of the bronchi.

We might, and perhaps ought, to have enlarged upon the truth and beauty of these delineations; upon the very valuable matter contained in the letter-press; and above all, on the general utility and great cheapness of this splendid work; but these qualities are conspicuous of themselves, and need not the assistance of our praise to be duly appreciated.

In conclusion, we have no hesitation in expressing our conviction that Dr. Carswell, by his researches and delineations, has thrown more light on the subject of tubercle than all that has been done before him; and that, in giving such a work to the public, at a rate which places it within the reach of every member of the profession, he is entitled in no small degree to the gratitude of his professional brethren.

